# Long-term treatment with testosterone alters ovary innervation in adult pigs

**DOI:** 10.1186/s13048-016-0273-4

**Published:** 2016-10-10

**Authors:** Barbara Jana, Karolina A. Meller, Michał Bulc, Jarosław Całka

**Affiliations:** 1 Division of Biology Reproduction, Institute of Animal Reproduction and Food Research of the Polish Academy of Sciences, Olsztyn, 10-748 Poland; 2 Division of Clinical Physiology, Faculty of Veterinary Medicine, University of Warmia and Mazury, Olsztyn, 10-718 Poland

**Keywords:** Ovary, Follicles, Innervation, Hyperandrogenism, Gilts

## Abstract

**Background:**

Intraovarian distribution and density of nerve fibres immunoreactive (IR) to protein gene product 9.5 (PGP 9.5) and containing dopamine-β-hydroxylase (DβH), neuropeptide Y (NPY), somatostatin (SOM), galanin (GAL) were determined.

**Methods:**

From day 4 of the first oestrous cycle to day 20 of the second studied cycle, experimental gilts (*n* = 3) were injected with testosterone (T), while control gilts (*n* = 3) received corn oil.

**Results:**

After T administration the numbers of fibres IR to PGP 9.5 and fibres IR to DβH, NPY and SOM were decreased. Fewer PGP 9.5- and DβH-IR terminals were observed within the ground plexus and around arteries and medullar veins, and medium tertiary follicles, and DβH-IR terminals in the vicinity of small tertiary follicles. T decreased the density of NPY-IR fibres in the medullar part of the ground plexus, and SOM-IR in the cortical part of the ground plexus.

**Conclusions:**

The obtained data show that long-term T treatment of gilts decreases the total number of intraovarian fibres, including sympathetic ones. These results suggest that elevated T levels that occur during pathological states may affect the innervation pattern of ovaries, and their function(s).

## Background

Hyperandrogenism is one of the most common and disturbing endocrine disorder of reproductive-aged women and can result from many pathological states. The ovary is an important source for androgen excess in women with polycystic ovary syndrome (PCOS) [1, 2] and androgen-secreting tumours [3]. Augmentation in the peripheral blood androgen levels is found in patients suffering from adrenal hyperplasia [4] and androgen-secreting tumours [5, 6]. Blood androgen levels are elevated in pigs with uterine inflammation [7] and polycystic ovaries [8], and in dogs with adrenal dysfunction [9].

The pig’s ovary receives its nerve supply from sympathetic, parasympathetic and sensory components of the peripheral nervous system (PNS). The sympathetic ovarian innervation derives from the caudal mesenteric ganglion (CaMG), sympathetic chain ganglia (SChGs; Th_10_-L_5_ and S_1_), ovarian and aorticorenal ganglia, as well as from the cranial part of the paracervical ganglion (PCG). The last-mentioned ganglion is also the source of parasympathetic neuronal inputs to the ovary. The sensory ovary-projecting neurons (referred to further as ovarian perikarya or ovarian neurons) occur in the dorsal root ganglia (DRGs) neuromers from Th10 to L5 [10]. Intraovarian sympathetic nerve fibres, constituting the most numerous population, are localized around follicles in all stages of development, the corpora lutea (CL), blood vessels and the interstitial gland, as well as within the ground plexus. These fibres, besides catecholamines (mainly noradrenaline - NA), may also express and release other active substances, for example, neuropeptide Y (NPY), somatostatin (SOM) and galanin (GAL) [10, 11]. NA and the above-mentioned peptides were found to influence steroidogenesis in ovarian cells [12–16]. Moreover, NA, NPY and GAL, acting by specific receptors or modulating the release of co-localized substances from nerve fibres, affect the blood flow and steroidogenesis in ovaries [17–19]. NA also affects ovulation [20], while NA [[21] Curry], SOM [22] and GAL [23] may influence follicular development.

Previous studies mainly show the effects of oestrogens on ovarian innervation. An increase in the content of NA was found in the ovaries of adult rats after injection of oestradiol valerate (EV; long-acting oestrogen), which was accompanied by morphological changes in gonads [24–26]. In turn, prenatal exposure to diethylstilbestrol, long-acting oestrogen, in newborn rats resulted in a drop in the density of the intraovarian sympathetic nerve network and a disruption in follicular maturation [27], as well as a reduction in the number of sympathetic ovarian perikarya in the celiac ganglion (CG) [28]. Lakomy et al. [29, 30] revealed a rise in the content of NA and the activity of acetylcholinesterase in the ovaries of oestradiol-17β (E_2_)-treated prepubertal gilts, as well as a decrease in the values of these parameters after the application of E_2_ together with progesterone (P_4_). We reported that long-term treatment of gilts with E_2_ changes the morphology and chemical coding of ovarian perikarya in the SChGs [31], CaMG [32], DRGs [33] and PCG [34]. Moreover, E_2_ exposure of gilts increases the total number of intraovarian fibres, including sympathetic ones [35].

Our knowledge on androgen influence on the PNS neurons supplying the female reproductive tract, including ovaries, is limited. In rats, during the late pregnancy [36] and after parturition [37], androstenedione (A_4_), may mediate a luteotropic effect acting on the CG neurons. In fact, the pig, due to its embryological, anatomical and physiological similarity to humans, constitutes an especially valuable species for bio-medical research, including that of ovary functions [38, 39]. Our earlier studies revealed that long-term testosterone (T) treatment alters the morphological and chemical organization of the CaMG [40], PCG [41] and SChGs [42] ovarian perikarya in adult gilts. Taking into consideration the above-mentioned findings, we hypothesize that elevated levels of androgens during pathological states may also affect the innervation pattern, including sympathetic ones, in the ovaries, and finally the gonadal functions. Therefore, we examined the ovaries of sexually mature gilts to determine the effect of long-term T administration on: 1) the total number and distribution of nerve fibres (stained for a general pan-neuronal marker - protein gene product 9.5- PGP 9.5), 2) the distribution and density of nerve fibres containing DβH and/or NPY, SOM, GAL, and 3) the populations of DβH-, NPY-, SOM- and GAL-immunoreactive (IR) nerve fibres in relation to the total number of PGP 9.5-IR nerve fibres.

## Methods

### Animals

The study was carried out on 6 crossbred gilts (Large White x Landrace), aged 7–8 months and weighing 90–110 kg, having had two controlled consecutive oestrous cycles. Behavioural oestrus was detected using a boar. Three days before surgical operations the gilts were transported from a farm to a local animal house and kept in individual stalls under natural light and temperature (April, May). They were fed a commercial grain mixture and tap water *ad libitum*.

### Experimental procedures

On day 3 of the first studied oestrous cycle (day 0 of the study), after induction of general anaesthesia by azaperone (2 mg/1 kg of body mass, Stresnil, Janssen Pharmaceutica N.V., Belgium) and sodium pentobarbital (30 mg/1 kg of body mass, Vetbutal, Biovet, Poland), a polyvinyl cannula (outer diameter 2.2 mm, inner diameter 1.8 mm, Tomel, Tomaszów Maz., Poland) was inserted into the jugular vein of each gilt in order to collect blood samples.

Next, the gilts were randomly assigned to one of two following groups: the control (group I, *n* = 3) and experimental (group II, *n* = 3). In the gilts of group I, from day 4 of the first studied oestrous cycle (day 1 of the study) to the expected day 20 of the second studied cycle, *i.e.*, within 38 consecutive days, 2 ml of oil was injected *i.m.* every 12 h (h; at 07:00 and 19:00 h). In turn, in the gilts of group II, at the same time and in the same manner 1000 μg of T (catalog no. 35800, Serva Electrophoresis GmbH, Germany) in 2 ml of corn oil was injected. The applied dose of T was determined based on our preliminary experiment, showing that its application increases the peripheral blood T concentration about 3.5 fold. According to available reports, about a 3- and 5-fold increase in the total T and bioavailable T, respectively, in blood concentrations accompanies adrenal hyperplasia [4], while the free androgen index is about 5-fold higher in women with PCOS than in controls [1]. For estimation of T, A_4_, E_2_, oestrone (E_1_) and P_4_ levels blood samples were collected from gilts of both groups through the whole period of T/oil injection (twice a day - 09:00 and 21:00 h). The samples were then immediately placed in an ice bath, where they were kept until centrifugation (10 min, 1,500 × g, at 4 °C). The plasma was decanted and stored at −20 °C until further processing. The analysis of androgen and oestrogen concentrations in the peripheral blood of the gilts was described earlier [40]. After the last blood sample collection the gilts were slaughtered by electric shock (ENZ 300 Metalowiec, Bydgoszcz, Poland) and both ovaries from each gilt were immediately dissected out and weighed. Afterwards, the volume, length, width and height of the gonads, as well as the number of follicles were estimated. The follicles were divided into three size classes: 1–3, 4–6 and 7–10 mm in diameter. Following the inspection of the ovarian surfaces, for immunocytochemical studies, ovaries were cut into 3 parts (two lateral and the third middle - containing the hilar region), and fixed by immersion in Zamboni’s fixative for 30 min, washed with 0.1 M phosphate buffer (PB; pH 7.4) over two days, and finally transferred to and stored at 4 °C in 18 % buffered sucrose solution (pH 7.4) containing 0.01 % natriumazide (NaN_3_) until further processing.

### Immunofluorescent procedures

To investigate the distribution and density of PGP 9.5-, DβH-, NPY-, SOM- and GAL-IR intraovarian nerve fibres, from every third part of ovary 9 (12-μm-thick) serial sections were cut in a cryostat (Frigocut, Reichert-Jung, Nussloch, Germany). The sections were mounted on chrome alum-coated slides and then subjected to a routine double-immunofluorescence technique described by Majewski and Heym [43]. Briefly, after air-drying at room temperature for 45 min and rinsing in 0.1 M phosphate-buffered saline (PBS; pH 7.4; 3 × 10 min), the sections were incubated in a blocking buffer containing 10 % normal goat serum (MP Biomedicals, Solon, OH, USA), 0.1 M PBS, 0.1 % donkey serum (Abcam, Cambridge, UK), 1 % Triton X-100 (Sigma-Aldrich, St. Louis, MO, USA), 0.05 % Thimerosal (Sigma-Aldrich, St. Louis, MO, USA), and 0.01 % NaN_3_ for 1 h at room temperature to reduce non-specific background staining. Subsequently, after another wash in PBS (3 × 10 min), the sections were incubated overnight at room temperature with two different species-specific primary antisera raised against PGP 9.5 (mouse, 7863–2004, AbD Serotec, dilution 1:1000), as well as with DβH (rabbit, AB1585, Millipore, dilution 1:2000 and mouse, MAB308, Millipore, dilution 1:1000), NPY (rabbit, NA1233, Enzo Life Sciences International, Inc., dilution 1:4000), SOM (rabbit, 8330–0154, AbD Serotec, dilution 1:50), and GAL (rabbit, AB2233, Millipore, dilution 1:4000). Following subsequent rinsing in PBS (3 × 10 min), the sections were incubated with secondary antisera Alexa Fluor 488 (donkey anti-mouse, A21202, Invitrogene, USA, dilution 1:1000), Alexa Fluor 546 (donkey anti-mouse, A10036, Invitrogene, USA, dilution 1:1000), Alexa Fluor 488 (donkey anti-rabbit, A21206, Invitrogene, USA, dilution 1:1000), Alexa Fluor 546 (donkey anti-rabbit, A11010, Invitrogene, USA, dilution 1:1000) for 2 h at room temperature to visualize the antibody combinations: PGP 9.5/DβH, PGP 9.5/NPY, PGP 9.5/SOM, PGP 9.5/GAL, DβH/NPY, DβH/SOM and DβH/GAL. Next, the washed sections were coverslipped in carbonate-buffered glycerol (pH 8.6). Standard tests (preabsorption for the used antisera with the respective antigen at a concentration of 20–50 μg antigen/ml diluted antiserum, omission of primary or secondary antisera and replacement by non-immune sera of all the primary antisera used) were employed to control the specificity of immunofluorescence. Also, DβH, NPY, SOM and GAL staining in the porcine CaMG ovary supplying neurons were applied as positive controls (data not shown). The immunocytochemical staining procedure for one combination of examined substances was conducted on nine randomly chosen ovarian sections from every one-third part of the organ derived from each studied animal.

Double-immunolabeled nerve fibres were analyzed and photographed under an Olympus BX51 microscope equipped with epifluorescence and the appropriate filter sets for FITC (B1 module, excitation filter 450–480 nm, barrier filter 515 nm) and CY3 (G1 module excitation filter 510–550 nm, barrier filter 590 nm). Pictures were captured by a digital camera connected to a PC and analyzed with the AnalySIS software (version 3.02, Olympus Soft Imaging Solutions, Muenster, Germany). In our study the distribution and density of PGP 9.5-, DβH-, NPY-, SOM- and GAL-IR intraovarian nerve fibres were estimated within the ground plexus and around follicles, blood vessels and interstitial glands. All stained processes identified in the surrounding zone of the above-mentioned structures were counted. Follicles, depending on the stage of development, were classified microscopically according to Wulff et al. [44] and Barboni et al. [45]: primordial - without granulosa cells, primary - surrounded by a single layer of cuboidal granulosa cells, secondary - with two or more granulosa cell layers without the antral cavity, tertiary - with antrum. Additionally, the tertiary follicles were divided into three size subclasses: small (to 3 mm in diameter), medium (4–6 mm in diameter) and large (7–10 mm in diameter). The diameter of follicles was measured using Microimage software (Olympus Polska sp. z o. o.,Warsaw, Poland).

### Statistical analyses

Data concerning the weight, volume and measurements of the ovaries, follicle numbers, as well as the density of innervation gained from two ovaries from each gilt were averaged *per* ovary. The mean (±SEM) weight, volume and size of the ovaries, the number of ovarian structures, as well as the total number of PGP 9.5-IR and the absolute numbers of DβH-, NPY-, SOM-and GAL-IR nerve fibres were compared between the groups using Student’s *t*-test. To calculate the statistical significance of the mean (±SEM) numbers of PGP 9.5-, DβH-, NPY-, SOM- and GAL-IR nerve fibres, between the groups and within the same group, supplying the particular ovarian structures, one-way analysis of variance (ANOVA) followed by the Newman-Keuls test was performed. To indicate the differences in frequency of DβH-, NPY-, SOM-, GAL-IR nerve fibres occurrence in the total population of PGP 9.5-IR nerve fibres, the total number of the PGP-IR nerve population in each group was accepted as 100 %. The numbers of DβH-, NPY-, SOM-, GAL-IR nerve fibres were expressed as a percentage (mean) of the total population of PGP 9.5-IR nerve fibres. Then, the Newman-Keuls test was applied for calculating the statistical significance of mean differences (ANOVA, InStat Graph Pad, San Diego, CA). Differences with a probability of *P* < 0.05 were considered significant.

## Results

### Macroscopic evaluation of the ovaries

Compared to the controls, treatment with T caused in the ovaries a reduction in the number of small (1–3 mm in diameter; 10.2 ± 0.33 vs. 20 ± 0.57, *P* < 0.001; respectively) and medium (4–6 mm in diameter; 3.16 ± 0.33 vs. 5.66 ± 0.6, *P* < 0.05; respectively) follicles, as well as a lack of large follicles (7–10 mm in diameter; 0 ± 0 vs. 4.4 ± 0.16, respectively). The weight and volume, as well as the length, width and height of the ovaries did not differ significantly in the control and experimental gilts (4.66 ± 0.29 vs. 3.51 ± 0.32 g; 4.79 ± 0.39 vs. 3.5 ± 0.29 ml; 1.4 ± 0.09 vs. 1.7 ± 0.08 cm; 2.91 ± 0.17 vs. 2.1 ± 0.29 cm; 2 ± 0.09 vs. 1.2 ± 0.28 cm, respectively).

### The distribution and density of PGP 9.5-IR nerve fibres in the ovaries

In the ovaries of gilts treated with T, the total number of PGP 9.5-IR nerve fibres was lower (*P* < 0.001) than in the control ovaries (160.23 ± 4.2 vs. 213.29 ± 3.72, respectively).

**Fig. 1:**
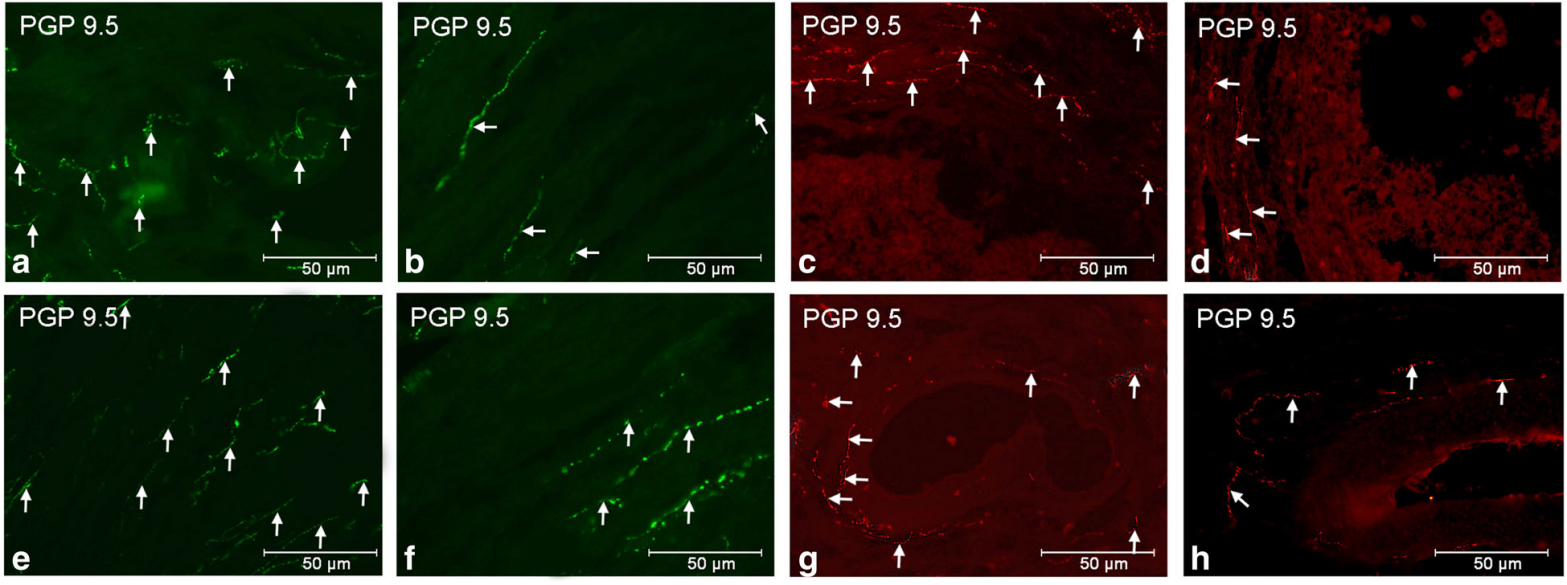
Ovarian PGP 9.5-IR nerve fibres (↑) in the control and T-treated gilts. In the control ovaries numerous nerve fibres visible within the cortical (**a**) and medullar (**e**) parts of the ground plexus, and around the medullar artery (**g**) and medium tertiary follicle (**c**). Note a drop in the density of PGP 9.5-IR nerve fibres after T administration within cortical (**b**) and medullar (**f**) parts of the ground plexus, and in the vicinity of the medullar artery (**h**) and medium tertiary follicle (**d**)

Compared to the control group (Fig. 1a, c, e, g), the T treatment led to a drop in the number of PGP 9.5-IR intraovarian nerve fibres in the cortical (*P* < 0.001, Fig. 1b) and medullar (*P* < 0.001, Fig. 1f) parts of the ground plexus, the near cortical (*P* < 0.001) and medullar (*P* < 0.001, Fig. 1h) arteries and medullar veins (*P* < 0.001), as well as medium tertiary follicles (*P* < 0.001, Fig. 1d). The distribution and density of PGP 9.5-IR nerve fibres supplying the ground plexus, follicles, the interstitial gland and blood vessels in both examined groups are depicted in Table 1.

**Table 1:** Mean (±SEM) number of PGP 9.5-IR nerve fibres in the ovaries of gilts

Ovarian structures		PGP 9.5	
		C	T
Cortex	Ground plexus	27.1 ± 0.29^a^	15.23 ± 0.44^a,x^
	Follicles:		
	*Primordial*	2.8 ± 0.2^b^	1.98 ± 0.39^b^
	*Primary*	2.69 ± 0.27^b^	2.29 ± 0.31^b^
	*Secondary*	7.3 ± 0.37^c^	8.27 ± 0.28^c^
	*Tertiary (mm):*		
	*- up to 3*	19.07 ± 0.73^d^	20.32 ± 0.91^d^
	*- 4–6*	28.25 ± 1^a^	16.68 ± 0.44^a,x^
	*- 7–10*	40.14 ± 1.25^e^	l.s.
	Arteries	14.27 ± 0.57^f^	7.32 ± 0.39^c,x^
	Veins	12.21 ± 0.6^f,g^	5.46 ± 0.51^c^
	Interstitial gland	5.2 ± 0.62^b,c,g^	6.48 ± 0.9^c^
Medulla	Ground plexus	19.29 ± 0.73^a^	10.55 ± 0.39^x^
	Arteries	19.64 ± 0.6^a^	10.72 ± 0.6^x^
	Veins	15.36 ± 0.55^b^	7.98 ± 0.33^x^

*C* control gilts, *T* testosterone-treated gilts; means with different superscriptions (a, b, c, d, e, f, g) indicate differences (*P* < 0.05–0.001) in the same group among particular structures in ovarian cortex or medulla; x - indicates differences (*P* < 0.001) between both groups for the same structure; l.s. - lack of structure

### Ovarian DβH-, NPY-, SOM-, GAL-IR nerve fibres as a percentage of PGP 9.5-IR nerve fibres

Long-term T administration led to a decrease (*P* < 0.01) in the number of ovarian DβH-IR nerve fibres (normalised against the total population of PGP 9.5-IR nerve fibres) compared with that in the control group (46.6 % vs 55.3 %, respectively). However, there was no significant difference between the control and T-injected groups in terms of NPY-IR (21.6 % vs 20.9 %, respectively), SOM-IR (14.9 % vs 12.4 %, respectively) and GAL-IR (9.4 % vs 8.8 %, respectively) fibres normalised against the total population of PGP 9.5-IR nerve fibres.

### The distribution and density of DβH-, NPY-, SOM- and GAL-IR nerve fibres in the ovaries

In the ovaries of T-treated gilts the absolute numbers of DβH-, NPY- and SOM-IR nerve fibres were lower than those calculated in the control group (74.71 ± 4.67 vs. 117.80 ± 2.1, *P* < 0.01; 31.89 ± 2.14 vs. 46.12 ± 1.78, *P* < 0.01; 19.9 ± 2.7 vs. 31.9 ± 0.91, *P* < 0.05; respectively). In turn, the absolute numbers of GAL-IR nerve terminals were similar in the control and T-treated gilts (20.25 ± 0.9 vs. 14.2 ± 2.69, respectively).

**Fig. 2:**
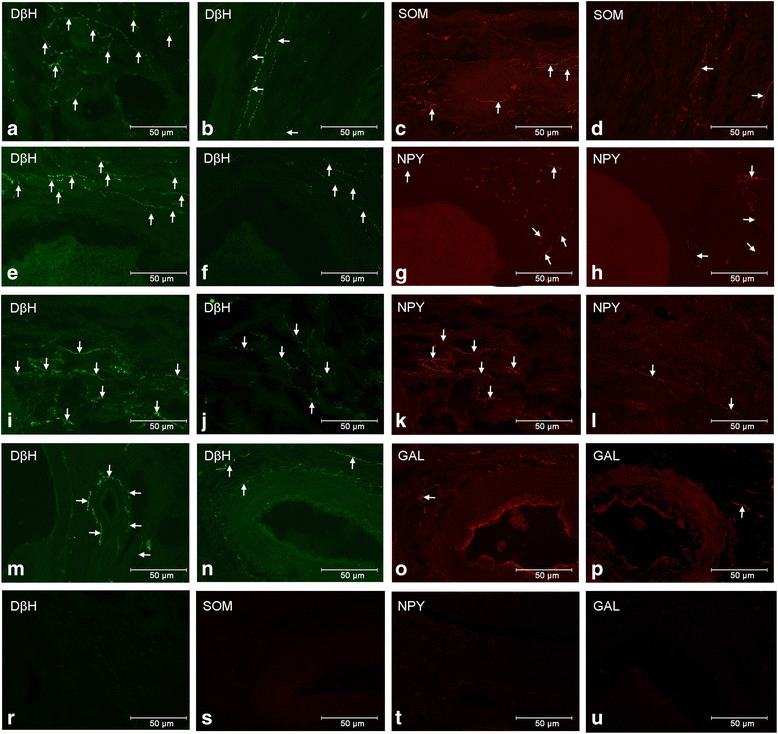
Ovarian DβH- and/or NPY-, SOM-, GAL-IR nerve fibres (↑) in the control and T-treated gilts. Within the cortical ground plexus of the control gilts numerous DβH- (**a**) and not numerous SOM-(**c**) IR nerve fibres are visible, while in the gilts receiving T anoticeable decrease in the population of DβH- (**b**) and SOM- (D) IR fibres is present. In the control ovary numerous DβH-IR fibres near medium tertiary follicle (**e**). Note a drop in the number of these nerve fibres in the T-injected gilt (**f**). Around medium tertiary follicles of the control (**g**) and T-treated (**h**) gilts the population of NPY-IR nerve fibres are similar. Within the medullar ground plexus of the control gilts numerous DβH- (**i**) or not numerous NPY- (**k**) IR nerve fibres are present. In turn, after Ttreatment, a decrease in the number of DβH- (**j**) and NPY- (**l**) immunoreactivity is visible. A greater population of DβH-IR fibres is present in the vicinity of the medullar artery in the control gilt (**m**) than after T treatment (**n**). The numbers of GAL-IR processes around medullar arteries of the control (**o**) and T-injected (**p**) gilts are similar. Negative controls for DβH (**r**), SOM (**s**), NPY (**t**) and GAL (**u**)

After T injections populations of the DβH-IR nerve fibres were lower within the cortical (*P* < 0.001, Fig. 2b) and medullar (*P* < 0.001, Fig. 2j) parts of the ground plexus, around small (*P* < 0.001) and medium (*P* < 0.001, Fig. 2f) tertiary follicles, as well as cortical (*P* < 0.001) and medullar (*P* < 0.001, Fig. 2n) arteries and medullar veins (*P* < 0.001) than in the control group (Figs. 2a, e, i, m). In the ovaries of T-injected gilts a decrease (*P* < 0.01) in the number of NPY-IR nerve terminals was found in the area of the medullar ground plexus (Fig. 2l) compared to the control group (Fig. 2k). In turn, the population of SOM-positive nerve fibres was lower (*P* < 0.001) within the cortical part of the ground plexus following T administration (Fig. 2d) than in the control ovaries (Fig. 2c). The application of T did not significantly change the innervation pattern of particular ovarian structures by GAL-IR nerve fibres (Figs. 2o, p). The distribution and density of DβH-, NPY-, SOM- and GAL-IR nerve fibres supplying the ground plexus, follicles, the interstitial gland and blood vessels in both studied groups are given in Table 2.

**Table 2:** Mean (±SEM) number of DβH-, NPY-, SOM- and GAL-IR nerve fibres in the ovaries of gilts

Ovarian structures		DβH		NPY		SOM		GAL	
		C	T	C	T	C	T	C	T
Cortex	Groundplexus	14.33 ± 1.93^a,b^	5.91 ± 0.33^a,x^	4.75 ± 0.38^a^	1.61 ± 0.05^a,c^	4.33 ± 0.17^a^	1.62 ± 0.02^a,b,x^	1.6 ± 0.48^a^	0 ± 0^a,b^
	Follicles:								
	*Primordial*	1.16 ± 0.2^c^	0.9 ± 0.07^b^	0.66 ± 0.22^b^	0.25 ± 0.06^a^	0.33 ± 0.22^b^	0 ± 0^c^	0.5 ± 0.19^a,b^	0.1 ± 0.03^a,b^
	*Primary*	2.44 ± 0.29^c^	1.8 ± 0.14^b^	0.33 ± 0.22^b^	0.35 ± 0.19^a^	0 ± 0^b^	0.91 ± 0.05^b,c^	0 ± 0^b^	0 ± 0^a,b^
	*Secondary*	3.72 ± 0.27^c^	6.31 ± 0.15^a^	1.8 ± 0.6^b,c^	2.27 ± 0.05^c,d^	1.4 ± 0.25^c^	2.03 ± 0.07^a^	0.4 ± 0.16^a,b^	0 ± 0^a,b^
	*Tertiary (mm):*								
	*- up to 3*	11.2 ± 1.2^a^	4.12 ± 0.33^a,b,x^	2.5 ± 0.02^c^	1.12 ± 0.05^a,d^	3.25 ± 0.31^d^	2.16 ± 0.25^a^	1.33 ± 0.68^a^	2.82 ± 0.54^c^
	*- 4–6*	16.8 ± 0.17^b^	5.73 ± 0.33^a,x^	4.25 ± 0.51^a^	2.28 ± 0.35^c,d^	3 ± 0.7^d^	1.58 ± 0.21^a,b^	2.75 ± 0.37^c^	2.9 ± 0.29^c^
	*- 7–10*	24.75 ± 1.21^d^	l.s.	7.25 ± 0.37^d^	l.s.	4.33 ± 0.29^a^	l.s.	3.5 ± 0.32^c^	l.s.
	Arteries	7.33 ± 0.59^e^	2.1 ± 0.06^b,x^	5 ± 0.68^a^	6 ± 0.05^b^	0.5 ± 0.19^b,c^	0.1 ± 0.06^c^	1 ± 0.27^a,b^	0.6 ± 0.03^a^
	Veins	4.14 ± 0.34^c,e^	1.35 ± 0.05^b^	5 ± 0.28^a^	2.1 ± 0.08^c,d^	1.5 ± 0.32^c^	0.5 ± 0.05^c^	1.33 ± 0.22^a^	0.41 ± 0.04^a,b^
	Interstitial gland	3.66 ± 0.4^c^	2.53 ± 0.33^a,b^	0.6 ± 0.22^b^	1.59 ± 0.06^a,d^	0 ± 0^b^	0 ± 0^c^	0 ± 0^b^	0 ± 0^b^
Medulla	Ground plexus	8 ± 0.33^a^	3.15 ± 0.08^x^	5.66 ± 0.17^a^	1.92 ± 0.06^a,x^	2.75 ± 0.31^a,b^	0.79 ± 0.01	1.8 ± 0.33^a^	1.6 ± 0.16^a^
	Arteries	2 ± 0.5^b^	4.3 ± 0.06^x^	3.25 ± 0.3^b^	1.12 ± 0.07^a^	3 ± 0.8^a^	0.8 ± 0.05	1.6 ± 0.3^a^	0.5 ± 0.08^b^
	Veins	8.6 ± 0.5^a^	3.17 ± 0.1^x^	4.5 ± 0.42^c^	3.36 ± 0.15^b^	1.6 ± 0.4^b^	1.9 ± 0.32	0.4 ± 0.16^b^	0 ± 0^b^

*C* control gilts, *T* testosterone-treated gilts; means with different superscriptions (a, b, c, d, e) indicate differences (*P* < 0.05–0.001) for particular substances in the same group among particular structures in ovarian cortex or medulla; x - indicates differences (*P* < 0.01, *P* < 0.001) for particular substances between both groups for the same structure; l.s. – lack of structure

### The patterns of co-localization of DβH with/or NPY, SOM, GAL in nerve fibres in the ovaries

In the ovaries of the gilts receiving T, compared to the control ovaries, all DβH-IR nerve fibres in the vicinity of secondary follicles were simultaneously NPY-IR, while DβH with NPY was co-localized in the part of fibres supplying cortical blood vessels. Following T administration, in the cortical part of ovaries the appearance of SOM expression in the part of DβH-IR nerve fibres around arteries, and a lack of co-localization of these substances near veins were found. The co-expression of DβH with GAL did not differ in fibres around primordial and tertiary small and medium follicles and cortical blood vessels in both groups. Similarly, in the nerve fibres occurring within the medullar ground plexus and in the vicinity of blood vessels of the experimental and control ovaries the co-expression DβH with/or NPY, SOM, GAL was similar. The co-localization patterns of DβH with/or NPY, SOM, GAL in nerve fibres innervating ground the plexus, follicles, the interstitial gland and blood vessels in both studied groups are presented in Table 3.

**Table 3:** The co-localization of DβH and/or NPY, SOM, GAL in the intraovarian nerve fibres of gilts

Ovarian structures		Group	DβH	NPY	SOM	GAL
Cortex	Ground plexus	C	-	+	+	-
		T	-	+	+	l.f.
	Follicles:					
	*Primordial*	C	-	-	-	-
		T	-	-	l.f.	-
	*Primary*	C	-	++	l.f.	l.f.
		T	-	++	+	l.f.
	*Secondary*	C	-	+	+	-
		T	-	++	+	l.f.
	*Tertiary (mm):*					
	*- up to 3*	C	-	+	+	+
		T	-	+	+	+
	*- 4–6*	C	-	+	+	+
		T	-	+	+	+
	*- 7–10*	C	-	+	+	+
		T	l.s.	l.s.	l.s.	l.s.
	Arteries	C	-	++	-	-
		T	-	+	+	-
	Veins	C	-	++	+	+
		T	-	+	-	+
	Interstitial gland	C	-	+	l.f.	l.f.
		T	-	+	l.f.	l.f.
Medulla	Ground plexus	C	-	+	+	+
		T	-	+	+	+
	Arteries	C	-	+	+	+
		T	-	+	+	+
	Veins	C	-	+	-	-
		T	-	+	-	-

*C* control gilts, *T* testosterone-treated gilts; co-localization of DβH with NPY, co-localization of DβH with SOM, co-localization of DβH with GAL in the part (+) or all (++) of nerve fibres; (−) lack of co-localization; *l.f.* lack of fibres, *l.s.* lack of structure

## Discussion

Our study shows that the long-term exposure of adult gilt ovaries to T resulted in disturbances in the development of follicles, a drop in the total population of nerve fibres (PGP 9.5-IR), including the total population of DβH-IR, as well as in the absolute numbers of NPY- and SOM-IR nerve fibres. Changes in the distribution and/or density of these fibres depending on the kind of chemical coding of the fibre and/or ovarian structure were also observed.

The peripheral blood androgen and oestrogen concentrations in gilts used in the current study have been reported earlier [40]. In T-injected gilts, in comparison to the control gilts, an increase in the T concentrations on all days of the study (approximately 3.5 fold), except for days 2, 12 and 35–37 was revealed. After T administration the mean daily concentrations of this steroid varied between 50 ± 13.6 and 141 ± 16.5 pg/ml. In T-treated gilts the levels of E_2_ were higher (approximately 1.6 fold) than in the control group on study days 4–14 and 21–29. In turn, mean daily E_2_ concentrations ranged from 3.2 to 16.8 ± 1.5 pg/ml. Following T injections the E_1_ levels decreased on days 8–11, 15, 16, 18, 19, 29–32, 37 and 38, while the A_4_ levels were significantly unchanged on all of the study days. Moreover, compared with the control group, mean P_4_ concentrations were lower in T-administered gilts on days 6–13 (ranged from 0.6 ± 0.1 to 3.4 ± 0.18 ng/ml), 22 (0.6 ± 0.07 ng/ml), 23 (0.4 ± 0.06 ng/ml) and 26–34 (ranged from 0.4 ± 0.04 to 0.8 ± 0.13 ng/ml) of the study [Jana et al. unpublished observations]. P_4_ concentrations allow the supposition that after T application the P_4_ synthesis in the corpora lutea was reduced in the first studied oestrous cycle, and the development of the corpora lutea did not occur in the second studied cycle. The disturbances in the course of the oestrous cycle were further confirmed by macroscopic examination of the ovaries, conducted on day 38 of the study (the expected day 20 of the second studied cycle), which revealed a drop in the number of small and medium tertiary follicles, and a lack of large tertiary follicles. These findings are congruent with previous studies performed on gilts receiving E_2_ for a long time [35]. The follicular development in the offspring of rats was also found to be suppressed after prenatal exposure to diethylstilbestrol [27]. The morphological changes in the ovaries of T-injected gilts (present study) were a consequence of long-term enhancement of T and E_2_ levels in the peripheral blood. It can be assumed that the effect of endogenous steroids on the ovaries of the T-injected gilts was in significant, which was confirmed by their decreased contents in gonadal tissue [Jana et al. unpublished observations]. We propose that changes in the morphology of ovaries and their lesser steroidogenic activity may result from a significant inhibition of the hypothalamic-pituitary axis function by higher E_2_ and T levels [46–48].

We found that T injections in the gilts resulted in a decrease of the total population of ovarian nerve fibres, demonstrated by PGP 9.5 immunoreactivity. Similarly, both single and repeated (*via* 21 days) E_2_ administration led to a decrease in the total population of PGP 9.5-IR terminals in the mouse uterus [49] and rat vagina [50]. In contrast, the greater populations of these fibres were visible in the ovaries of the long-term E_2_-treated gilts [35]. A rise in the number of PGP 9.5-IR fibres was also observed in the mammary gland [51] and earlobe [52] in adult ovariectomized (OVX) rats after short-term (*via* 7 days) E_2_ exposure. Data exist showing that dehydroepiandrosterone (DHEA) exerted a stimulatory effect on nerve density in OVX rat vagina through an androgenic action [53]. These discrepancies in androgen and oestrogen effects on the population of nerve fibres are probably due to species differences, the kind of organ, as well as the time and doses of administered steroid hormones.

In the present study, in the ovaries of gilts treated with T, the population of DβH-IR nerve fibres, calculated in relation to the total population of PGP 9.5 fibres as well as the absolute number of DβH-IR fibres, markedly decreased. T treatment resulted in a drop in the absolute number of the NPY- and SOM-IR nerve fibres. In turn, populations of GAL nerve fibres (total and absolute) were similar in both studied groups. We also found that in the ovaries of T-treated gilts the distribution and/or density of fibres positive for DβH, NPY and SOM depended on the kind of chemical coding of the fibre and/or ovarian structure. However, changes in the innervation of the gonads by DβH-IR nerve fibres referred only to their density around/within the particular structures but not their distribution. Existing data show that in T-treated rats renal tyrosine hydroxylase (TH) activity [54] decreased, and that in rats exposed to E_2_ the level of this enzyme in the superior cervical ganglia was reduced [55]. After E_2_ injections some ovarian structures were found to be supplied by higher numbers of DβH-IR fibres [35]. Similarly, Anesetti et al. [56] reported an increase in the number of intraovarian TH-IR nerve endings in immature rats in response to cypionate estradiol. Moreover, in the ovaries of adult rats a greater number of NA-ergic nerve fibres was found after a single EV injection [24, 25]. Also, E_2_ treatment in adult OVX rats led to an increase in the density of TH-positive nerve terminals in the mammary gland [51]. A higher population of noradrenergic nerve fibres in porcine ovaries was also observed on 30 day of pregnancy, when E_2_ level is the highest [57]. However, this steroid did not exert any significant effect on the density of TH-IR nerve endings in rat urethra [58] or vagina [59]. Applied in our study, T injections resulted in a drop in the number of NPY- or SOM-IR fibres within the medullar and cortical parts of the ground plexus, respectively. After hormonal treatment the GAL-IR fibres were not visible within the cortical part of the ground plexus or around secondary follicles, while these fibres appeared around primary follicles. In our earlier study, E_2_ injections resulted in an increase in the number of NPY- or GAL-IR fibres within the cortical and medullar parts of the ground plexus, respectively. This hormonal treatment induced a parallel increase in the density of NPY-IR fibres around medullar arteries, while SOM- and GAL-IR fibres were not found in the vicinity of primordial follicles [35]. It was also reported that short-term (*via* 7 days) E_2_ application increased the number of calcitonin gene-related peptide (CGRP)-IR fibres in the earlobe [52] and mammary gland [51] in adult OVX rats. However, the population of these endings was not markedly changed in rat uterus after E_2_ treatment [52]. There is a lack of data concerning the effect of androgens on the innervation patterns of ovaries by NPY-, SOM- and GAL-positive nerve fibres. The abundance of peptidergic (NPY, VIP) fibres in rat prostate during postnatal development was regulated by androgens [60].

In the ovaries of T-treated gilts we also revealed changes in the co-localization patterns of DβH with/or NPY, SOM in nerve fibres. There are no data concerning the co-localization of neurotransmitters in the intraovarian nerve fibres in response to androgen administration. However, it is important to add that the changes in co-localization patterns observed in the present study are similar to those revealed in the ovaries of E_2_-treated gilts [35].

It is difficult to indicate the mechanism(s) underlying the drop in the total number of nerve fibres, as well as the subsets of DβH-, NPY- and SOM-IR nerve fibres in the ovaries of gilts receiving T. We suppose that these changes may be associated with the reduction of perikarya within the ganglia innervating ovaries. We reported earlier that in the CaMG [40], SChGs [42] and PCG [41] of T-treated gilts, from which ovaries were obtained for the present study, down-regulation of the total number of ovarian perikarya was found. Moreover, fewer populations of DβH-, NPY- and SOM-IR fibres in the ovaries of T-injected gilts were coincident with decreased populations of ovarian perikarya expressing these substances in the above-mentioned sympathetic ganglia [40, 42]. The lower number of SOM-IR found after T treatment (present study) also corresponds with the reduced population of ovarian perikarya possessing SOM in the PCG [41]. In addition, changes in the innervation pattern of gonads in the gilts receiving T might result from T and E_2_ effects on other sympathetic peripheral ganglia supplying the ovaries [10]. Available data show that the reduction in the set of the intraovarian sympathetic nerve terminals in rats prenatally exposed to diethylstilbestrol [27] was accompanied by a decrease in the number of sympathetic ovarian perikarya in the celiac ganglion [28]. It is possible that the lower density of intraovarian nerve fibres in the T-injected gilts may be connected with the decreased production of nerve growth factor (NGF), which, as is generally known, plays a crucial role in the development, survival and differentiation of sympathetic and sensory neurons, and in the regulation of axon and dendrite growth [61]. We propose that elevated T and E_2_ levels occurring in the blood of T-injected gilts, by the stimulation of AR [62] and ERs [63] in ovarian steroidogenic cells, could lead to lower NGF and/or its receptor expression in the gonads. It is known that T suppresses NGF production in fibroblast cells [64] and NGF receptor mRNA expression in Sertoli cells [65]. Chronic treatment of rats with E_2_ reduced NGF protein content [55] and p75 NTR receptors expression [66] in the superior cervical ganglia. Due to the fact that in the current study aromatizable androgen has been applied to evoke hyperandrogenic state, it is impossible to precisely ascribe the observed changes to T or E_2_. Clarification of this question needs the application of non-aromatizable T - dihydrotestosterone (DHT).

The changes in the populations of ovarian nerve fibres in T-injected gilts revealed in the present study may have an importance for gonadal functions. A drop in the numbers of fibres expressing DβH within the ground plexus, near blood vessels and follicles, and/or NPY (transmitter occurring in the greatest number of sympathetic nerve fibres) within the ground plexus may cause disturbances in a variety of ovarian sympathetic actions, including regulation of steroidogenesis and blood supply, and in relation to DβH-IR fibres also in the regulation of follicular development. This supposition is based on previous studies demonstrating that under physiological conditions NA and NPY were found to increase synthesis of P_4_ and E_2_ in granulosa and luteal cells in humans [12] and many animal species [13–15], as well as to affect the blood flow in ovaries [17–19]. Moreover, the participation of NA in follicular development is well-known [21]. In turn, a reduction in the population of fibres containing SOM (transmitter occurring in sympathetic and parasympathetic fibres) found within the ground plexus in the ovaries of T-treated gilts may be significant for gonadal steroidogenesis [16] and follicular development [22].

## Conclusions

After long-term T administration in sexually mature gilts a decrease in the total population of intraovarian nerve fibres, including fibres containing DβH, NPY and SOM is observed. Moreover, our study suggests that hyperandrogenism may affect the innervation pattern of the ovaries and consequently their function(s). However, further studies determining the mechanism(s) underlying changes in the ovarian innervation pattern during hyperandrogenic states are necessary. In sum, the present findings support the significant role of steroid hormones modulating neuronal plasticity.

## Abbreviations

A4: Androstenedione
CaMG: Caudal mesenteric ganglion
CG: Celiac ganglion
CGRP: Calcitonin gene-related peptide
CL: Corpora lutea
DHEA: Dehydroepiandrosterone
DHT: Dihydrotestosterone
DRGs: Dorsal root ganglia
DβH: Dopamine-β-hydroxylase
E1: Oestrone
E2: Oestradiol-17β
EV: Estradiol valerate
GAL: Galanin
IR: Immunoreactive
NA: Noradrenaline
NaN3: Natriumazide
NGF: Nerve growth factor
NPY: Neuropeptide Y
OVX: Ovariectomized
P4: Progesterone
PB: Phosphate buffer
PBS: Phosphate-buffered saline
PCG: Paracervical ganglion
PCOS: Polycystic ovary syndrome
PGP 9.5: Protein gene product 9.5
PNS: Peripheral nervous system
SChGs: Sympathetic chain ganglia
SOM: Somatostatin
T: Testosterone
TH: Tyrosine hydroxylase

## References

[1] Panidis D, Farmakiotis D, Rousso D, Katsikis I, Kourtis A, Diamanti-Kandarakis E (2005). Serum luteinizing hormone levels are markedly increased and significantly correlated with Delta 4-androstenedione levels in lean women with polycystic ovary syndrome. FertilSteril.

[2] Keefe CC, Goldman MM, Zhang K, Clarke N, Reitz RE, Welt CK (2014). Simultaneous measurement of thirteen steroid hormones in women with polycystic ovary syndrome and control women using liquid chromatography-tandem mass spectrometry. PLoS One.

[3] Singh P, Deleon F, Anderson R (2012). Steroid cell ovarian neoplasm, not otherwise specified: a case report and review of the literature. Case Rep Obstet Gynecol.

[4] Goodarzi MO, Dawson DW, Li X, Lei Z, Shintaku P, Rao CV, Van Herle AJ (2003). Virilization in bilateral macronodular adrenal hyperplasia controlled by luteinizing hormone. J Clin Endocrinol Metab.

[5] Marcondes JA, Barcellos CR, Rocha MP, Bisi H (2011). Changing pattern of gonadotropins in a patient with an adrenal-androgen secreting tumor. Clinics (Sao Paulo).

[6] Varma T, Panchani R, Goyal A, Maskey R (2013). A case of androgen-secreting adrenal carcinoma with non-classical congenital adrenal hyperplasia. Indian J Endocrinol Metab.

[7] Jana B, Kucharski J, Ziecik AJ (2004). Effect of intrauterine infusion of Escherichia coli on hormonal patterns in gilts during the oestrous cycle. Reprod Nutr Dev.

[8] Szulańczyk-Mencel K, Rzasa A, Bielas W (2010). Relationships between ovarian cysts and morphological and hormonal state of ovarian cortex in sows. Anim Reprod.

[9] Hill KE, Scott-Moncrieff JC, Koshko MA, Glickman LT, Glickman NW, Nelson RW, Blevins WE, Oliver JW (2005). Secretion of sex hormones in dogs with adrenal dysfunction. J Am Vet Med Assoc.

[10] Majewski M (1997). Afferent and efferent innervation of the porcine ovary-sources of origin and chemical coding. Acta Acad Agric Tech Olst, Veterinaria.

[11] Jana B, Majewski M (2007). Influence of the peripheral nervous system on ovarian function. Medycyna Wet.

[12] Barreca A, Valli B, Cesarone A, Arvigo M, Balasini M, Battista La Sala G, Garrone S, Minuto F, Giordano G (1998). Effects of the neuropeptide Y on estradiol and progesterone secretion by human granulosa cells in culture. Fertil Steril.

[13] Sosa ZY, Casais M, Rastrilla AM, Aguado L (2000). Adrenergic influences on coeliac ganglion affect the release of progesterone from cycling ovaries: characterization of an in vitro system. J Endocrinol.

[14] Miyamoto A, Brückmann A, von Lützow H, Schams D (1993). Multiple effects of neuropeptide Y, substance P and vasoactive intestinal polypeptide on progesterone and oxytocin release from bovine corpus luteum in vitro. J Endocrinol.

[15] Pitzel L, Jarry H, Wuttke W (1991). Effects of substance-P and neuropeptide-Y on in vitro steroid release by porcine granulosa and luteal cells. Endocrinology.

[16] Andreani CL, Lazzarin N, Pierro E, Lanzone A, Mancuso S (1995). Somatostatin action on rat ovarian steroidogenesis. Hum Reprod.

[17] Xu XJ, Hao JX, Wiesenfeld-Hallin Z, Håkanson R, Folkers K, Hökfelt T (1991). Spantide II, a novel tachykinin antagonist, and galanin inhibit plasma extravasation induced by antidromic C-fiber stimulation in rat hindpaw. Neuroscience.

[18] Markiewicz W, Jaroszewski JJ, Barszczewska B, Sienkiewicz W (2003). Localization of neuropeptide Y and norepinephrine in the porcine ovarian artery and their influence on the local blood pressure. Folia Histochem Cytobiol.

[19] Keator CS, Custer EE, Hoagland TA, Schreiber DT, Mah K, Lawsom AM, Slayden OD, McCraken JA (2010). Evidence for a potential role of neuropeptide Y in ovine corpus luteum function. Dom Anim Endorcinol.

[20] Traurig HH, Papka RE, Maggi CA (1993). Autonomic efferent and visceral sensory innervation of the female reproductive system: special reference to the functional roles of nerves in reproductive organs. Nervous control of the urogenital system.

[21] Curry TE, Lawrence IE, Burden HW (1984). Ovarian sympathectomy in the guinea pig. I. Effects on follicular development during the estrous cycle. Cell Tissue Res.

[22] Nestorović N, Manojlović-Stojanoski M, Ristić N, Sekulić M, Sošić-Jurjević B, Filipović B, Milošević V (2008). Somatostatin-14 influences pituitary-ovarian axis in peripubertal rats. Histochem Cell Biol.

[23] Crawley JN (1995). Biological actions of galanin. Regul Pept.

[24] Lara HE, Ferruz JL, Luza S, Bustamante DA, Borges Y, Ojeda SR (1993). Activation of ovarian sympathetic nerves in polycystic ovary syndrome. Endocrinology.

[25] Lara HE, Dissen GA, Leyton V, Paredes A, Fuenzalida H, Fiedler JL, Ojeda SR (2000). An increased intraovarian synthesis of nerve growth factor and its low affinity receptor is a principal component of steroid-induced polycystic ovary in the rat. Endocrinology.

[26] Rosa-E-Silva A, Guimaraes MA, Padmanabhan V, Lara HE (2003). Prepubertal administration of estradiol valerate disrupts cyclicity and leads to cystic ovarian morphology during adult life in the rat: role of sympathetic innervation. Endocrinology.

[27] Shinohara Y, Matsumoto A, Mori T (1998). Effects of prenatal exposure to diethylstilbestrol on the sympathetic nervous system in the rat ovary. Neurosci Lett.

[28] Shinohara Y, Matsumoto A, Hayashi S, Mori T (2000). Prenatal exposure to diethylstilbestrol decreases the number of estrogen receptor alpha-containing neurons innervating the ovary in rat celiac ganglion. Neuroscience.

[29] Lakomy M, Kaleczyc J, Całka J. The effect of oestradiolum benzoicum and progesterone on AChE activity in the nerves of the female reproductive system of immature pigs. Gegenbaurs Morphol Jahrb. 1986a;132:333–48. 3743996

[30] Lakomy M, Kotwica J, Całka J, Kaleczyc J. The effect of oestradiolum benzoicum and progesterone on the noradrenaline content in organs of the female reproductive system of sexually immature pigs. Gegenbaurs Morphol Jahrb. 1986b;132:129–43. 3710109

[31] Koszykowska M, Całka J, Gańko M, Jana B (2011). Long-term estradiol-17β administration reduces population of neurons in the sympathetic chain ganglia supplying the ovary in adult gilts. Exp Mol Pathol.

[32] Koszykowska M, Całka J, Szwajca P, Jana B (2011). Long-term estradiol-17β administration decreases the number of neurons in the caudal mesenteric ganglion innervating the ovary in sexually mature gilts. J Reprod Dev.

[33] Jana B, Lata M, Bulc M, Całka J (2012). Long term estradiol-17β administration changes population of the dorsal root ganglia neurons innervating the ovary in the sexually mature gilts. Neuropeptides.

[34] Jana B, Palus K, Czarzasta J, Całka J (2013). Long-term estradiol-17β administration changes population of paracervical ganglion neurons supplying the ovary in adult gilts. J Mol Neurosci.

[35] Koszykowska M, Całka J, Nidzgorska A, Jana B (2013). Exogenous long-term treatment with 17β-oestradiol alters the innervation pattern in pig ovary. Reprod Fertil Dev.

[36] Vallcaneras SS, Casais M, Delgado SM, Filippa V, Mohamed F, Sosa Z, Rastrilla AM (2009). Androgen receptors in coeliac ganglion in late pregnant rat. Steroids.

[37] Vallcaneras SS, Casais M, Anzulovich AC, Delgado SM, Sosa Z, Telleria CM, Rastrilla AM (2001). Androstenedione acts on the coeliac ganglion and modulates luteal function *via* the superior ovarian nerve in the postpartum rat. J Steroid Biochem Mol Biol.

[38] Verma N, Rettenmeier AW, Schmitz-Spanke S (2011). Recent advances in the use of Susscrofa (pig) as a model system for proteomic studies. Proteomics.

[39] Swindle MM, Makin A, Herron AJ, Clubb FJ, Frazier KS (2012). Swine as models in biomedical research and toxicology testing. Proc Natl Acad Sci U S A.

[40] Jana B, Rytel L, Czarzasta J, Całka J (2013). Reduction of the number of neurons in the caudal mesenteric ganglion innervating the ovary in sexually mature gilts following testosterone administration. J Neuroendocrinol.

[41] Jana B, Całka J, Bulc M, Czarzasta J (2014). Long-term testosterone administration affects the number of paracervical ganglion ovary-projecting neurons in sexually mature gilts. Neurosc Res.

[42] Jana B, Całka J, Rytel L, Czarzasta J (2015). Morphological and neurochemical characterization of the ovarian sympathetic chain ganglia perikarya in testosterone-treated sexually matured pigs. Ann Anat.

[43] Majewski M, Heym C (1991). The origin of ovarian neuropeptide Y (NPY)-immunoreactive nerve fibres from the inferior mesenteric ganglion in the pig. Cell Tissue Res.

[44] Wulff C, Wilson H, Wiegand SJ, Rudge JS, Fraser HM (2002). Prevention of thecal angiogenesis, antral follicular growth, and ovulation in the primate by treatment with vascular endothelial growth factor trap R1R2. Endocrinology.

[45] Barboni B, Martelli A, Berardinelli P, Russo V, Turriani M, Bernabò N, Lucidia P, Mattioli M (2004). Ovarian follicle vascularization in fasted pig. Theriogenology.

[46] Pieper DR, Gala RR, Schiff MA, Regiani SR, Marshall JC (1984). Pituitary gonadotropin-releasing hormone (GnRH) receptor responses to GnRH in hypothalamus-lesioned rats: inhibition of responses by hyperprolactinemia and evidence that testosterone and estradiol modulate gonadotropin secretion at postreceptor sites. Endocrinology.

[47] Ziecik AJ, Britt HJ, Esbenshade KL (1988). Short loop feedback of estrogen-induced luteinizing hormone surge in pigs. Endocrinology.

[48] Sullivan SD, Moenter SM (2005). GABAergic integration of progesterone and androgen feedback to gonadotropin-releasing hormone neurons. Biol Reprod.

[49] Zoubina EV, Smith PG (2001). Sympathetic hyperinnervation of the uterus in the estrogen receptor alpha knock-out mouse. Neuroscience.

[50] Ting AY, Blacklock AD, Smith PG (2004). Estrogen regulates vaginal sensory and autonomic nerve density in the rat. Biol Reprod.

[51] Blacklock AD, Smith PG (2004). Estrogen increases calcitonin gene-related peptide-immunoreactive sensory innervation of rat mammary gland. J Neurobiol.

[52] Blacklock AD, Cauveren JA, Smith PG (2004). Estrogen selectively increases sensory nociceptor innervation of arterioles in the female rat. Brain Res.

[53] Pelletier G, Ouellet J, Martel C, Labrie F (2013). Androgenic action of dehydroepiandrosterone (DHEA) on nerve density in the ovariectomized rat vagina. J Sex Med.

[54] Sharma U, Dunphy G, Ely D (2002). Testosterone increased blood pressure and decreased renal tyrosine hydroxylase activity in SHR/y and Wistar-Kyoto rats. Clin Exp Hypertens.

[55] Kaur G, Janik J, Isaacson LG, Callahan P (2007). Estrogen regulation of neurotrophin expression in sympathetic neurons and vascular targets. Brain Res.

[56] Anesetti G, Lombide P, Chávez-Genaro R (2009). Prepubertal estrogen exposure modifies neurotrophin receptor expression in celiac neurons and alters ovarian innervation. Auton Neurosci.

[57] Szatkowska C, Lakomy M (1987). Adrenergic innervation of swine ovaries in various periods of pregnancy. Pol Arch Weter.

[58] Smith PG, George M, Bradshaw S (2009). Estrogen promotes sympathetic nerve regeneration in rat proximal urethra. Urology.

[59] Pessina MA, Hoyt RF, Goldstein I, Traish AM (2006). Differential effects of estradiol, progesterone, and testosterone on vaginal structural integrity. Endocrinology.

[60] Rodríguez R, Pozuelo JM, Martín R, Arriazu R, Santamaria L (2005). Stereological quantification of nerve fibers immunoreactive to PGP 9.5, NPY, and VIP in rat prostate during postnatal development. J Androl.

[61] Huang EJ, Reichardt LF (2001). Neurotrophins: roles in neuronal development and function. Annu Rev Neurosci.

[62] Duda M, Slomczyńska M (2007). Immunohistochemical localization of androgen receptor in two subpopulations of porcine granulosa cells in vitro. Reprod Domest Anim.

[63] Knapczyk K, Duda M, Durlej M, Galas J, Koziorowski M, Slomczynska M (2008). Expression of estrogen receptor alpha (ERalpha) and estrogen receptor beta (ERbeta) in the ovarian follicles and corpora lutea of pregnant swine. Domest Anim Endocrinol.

[64] Siminoski K, Bernanke J, Kay C, Murphy RA (1986). Steroids and triiodothyronine reduce nerve growth factor concentrations in medium conditioned by L-929 fibroblasts. Endocrinology.

[65] Persson H, Ayer-Le Lievre C, Söder O, Villar MJ, Metsis M, Olson L, Ritzen M, Hökfelt T (1990). Expression of beta-nerve growth factor receptor mRNA in Sertoli cells downregulated by testosterone. Science.

[66] Hasan W, Smith HJ, Ting AY, Smith PG (2005). Estrogen alters trkA and p75 neurotrophin receptor expression within sympathetic neurons. J Neurobiol.

